# Human Body Composition and Immunity: Visceral Adipose Tissue Produces IL-15 and Muscle Strength Inversely Correlates with NK Cell Function in Elderly Humans

**DOI:** 10.3389/fimmu.2018.00440

**Published:** 2018-03-06

**Authors:** Ahmad Al-Attar, Steven R. Presnell, Jody L. Clasey, Douglas E. Long, R. Grace Walton, Morgan Sexton, Marlene E. Starr, Philip A. Kern, Charlotte A. Peterson, Charles T. Lutz

**Affiliations:** ^1^Department of Pathology and Laboratory Medicine, College of Medicine, University of Kentucky, Lexington, KY, United States; ^2^Department of Kinesiology and Health Promotion, College of Education, University of Kentucky, Lexington, KY, United States; ^3^Department of Rehabilitation Sciences, College of Health Sciences, University of Kentucky, Lexington, KY, United States; ^4^Department of Surgery, College of Medicine, University of Kentucky, Lexington, KY, United States; ^5^Division of Endocrinology, Department of Medicine, College of Medicine, University of Kentucky, Lexington, KY, United States; ^6^Department of Microbiology, Immunology, and Molecular Genetics, College of Medicine, University of Kentucky, Lexington, KY, United States

**Keywords:** natural killer cell, adipose tissue, IL-15, skeletal muscle strength, aging

## Abstract

Natural killer (NK) lymphocyte-mediated cytotoxicity and cytokine secretion control infections and cancers, but these crucial activities decline with age. NK cell development, homeostasis, and function require IL-15 and its chaperone, IL-15 receptor alpha (IL-15Rα). Macrophages and dendritic cells (DC) are major sources of these proteins. We had previously postulated that additional IL-15 and IL-15Rα is made by skeletal muscle and adipose tissue. These sources may be important in aging, when IL-15-producing immune cells decline. NK cells circulate through adipose tissue, where they may be exposed to local IL-15. The objectives of this work were to determine (1) if human muscle, subcutaneous adipose tissue (SAT), and visceral adipose tissue (VAT) are sources of IL-15 and IL-15 Rα, and (2) whether any of these tissues correlate with NK cell activity in elderly humans. We first investigated IL-15 and IL-15Rα RNA expression in paired muscle and SAT biopsies from healthy human subjects. Both tissues expressed these transcripts, but IL-15Rα RNA levels were higher in SAT than in skeletal muscle. We also investigated tissue obtained from surgeries and found that SAT and VAT expressed equivalent amounts of IL-15 and IL-15Rα RNA, respectively. Furthermore, stromal vascular fraction cells expressed more IL-15 RNA than did adipocytes. To test if these findings related to circulating IL-15 protein and NK cell function, we tested 50 healthy adults aged > 70 years old. Plasma IL-15 levels significantly correlated with abdominal VAT mass in the entire cohort and in non-obese subjects. However, plasma IL-15 levels did not correlate with skeletal muscle cross-sectional area and correlated inversely with muscle strength. Plasma IL-15 did correlate with NK cell cytotoxic granule exocytosis and with CCL4 (MIP-1β) production in response to NKp46-crosslinking. Additionally, NK cell responses to K562 leukemia cells correlated inversely with muscle strength. With aging, immune function declines while infections, cancers, and deaths increase. We propose that VAT-derived IL-15 and IL-15Rα is a compensatory NK cell support mechanism in elderly humans.

## Introduction

Natural killer (NK) cells are classified as members of the type 1 innate lymphoid cells (1). NK cells defend against infection, both directly and by orchestrating T cell, DC, monocyte, and macrophages (Mφ) responses (2). NK cells also may eliminate cancer cells and senescent cells (3, 4). Peripheral NK cells develop in the bone marrow and secondary lymphoid organs, where they are nurtured by multiple cell types and cytokines. IL-15, which is critical for mature NK cell development, homeostasis and function (5), signals *via* a trimeric receptor comprised of IL-15Rα, CD122, and CD132. IL-15 RNA is made in the bone marrow, secondary lymphoid tissues, and many nonlymphoid tissues, including skeletal muscle and adipose tissue. Although IL-15Rα is part of the IL-15 receptor, it also is required for IL-15 secretion and appearance on cell surfaces. In Mφ, DC, and other producing cells, IL-15 and IL-15Rα bind together with very high affinity. The complex is transported to the cell surface, where it stimulates neighboring NK cells in a paracrine fashion (5, 6). IL-15/IL-15Rα complexes also circulate to act on NK cells in an endocrine fashion (7). Two observations indicate that physiological IL-15 levels are dose-limiting for NK cells homeostasis: hemizygous IL-15 mice have low NK cell number and exogenous IL-15 boosts NK cell number in both normal mice and primates (8–10).

Human NK cells are classified into two major subsets based on their CD56 surface expression. Most circulating blood NK cells are CD56^dim^, while 5–15% are CD56^bright^. CD56^bright^ NK cells are poorly cytotoxic but secrete high levels of cytokines and chemokines in response to inflammatory cytokines. Although CD56^dim^ NK cells respond weakly to inflammatory cytokines, they kill target cells (such as the erythroleukemia cell line K562) and secrete chemokines and cytokines in response to antibody-coated cells and tumor cells.

Natural killer cell numbers are maintained in healthy elderly people, but NK-mediated cytotoxicity and secretion of immunoregulatory cytokines and chemokines decline with age (11, 12). Aging-related NK defects in mice are due, at least in part, to ineffective support from stromal cells (13–15). These defects could be due to decreased Mφ and dendritic cell IL-15 production and presentation (13, 15). Decreased NK cell activity in elderly people correlates with an increased incidence and severity of viral and bacterial infections and deaths (11, 16). Moreover, low NK function was found to be associated with increased cancer rates in subsequent years (17).

The objectives of this work were to determine (1) if human muscle, subcutaneous adipose tissue (SAT), and visceral adipose tissue (VAT) are sources of IL-15 and IL-15 Rα, and (2) whether any of these tissues correlate with NK cell activity in elderly humans. We found that IL-15 and IL-15Rα RNA are expressed in muscle, SAT, and VAT, but with relatively lower IL-15Rα RNA levels in skeletal muscle. Because skeletal muscle produces high levels of IL-15 RNA, we initially hypothesized that relatively strong elderly individuals would have higher IL-15 levels and more robust NK cell response (18). Contrary to our prediction, we found that plasma IL-15 level did not associate with lean tissue mass, but rather with VAT. Additionally, NK cell response inversely correlated with muscle strength.

## Materials and Methods

### Subjects

In accordance with the Declaration of Helsinki (modified in 2008), all protocols were approved by the Institutional Review Board of the University of Kentucky, Lexington, KY, USA. All subjects were made aware of the design and purpose of the studies, and all subjects signed consent forms. The cohorts are summarized in Table S1 in Supplementary Material, with additional information provided in some of the figure legends. Cohort A vastus lateralis muscle and SAT biopsies from healthy research subjects were frozen in liquid nitrogen and stored at −80°C. Cohort B SAT and VAT were obtained from discarded surgery specimens, immediately put on ice for no more than 3 h, and immediately processed into stromal vascular fraction (SVF) and adipocyte fractions or stored at −80°C. Cohort C VAT, including mesenteric fat, epiploic appendages, and omentum were obtained from discarded surgery specimens, immediately snap frozen in liquid nitrogen, and stored at −80°C. Cohort D blood samples were obtained between 9:30 a.m. and 12:45 p.m. and kept at room temperature until processing within 2 h of collection.

### Flow Cytometry and NK Cell Stimulation

As described in Ref. (19), whole blood was diluted with PBS and the mononuclear cells were recovered using Lymphoprep^®^ lymphocyte separation medium (Axis-Shield, Oslo, Norway). For antibody staining, ~0.5 × 10^6^ fresh mononuclear cells were washed and incubated with human IgG for 15 min at room temperature to block Fc-receptor binding and then stained on ice for 30 min with combinations of fluorescently labeled mAb, including those specific for CD3, CD16, and CD56 to allow for identification of CD56^bright^ and CD56^dim^ subsets (19). After washing, the cells were analyzed on a LSR-II flow cytometer (BD, San Jose, CA, USA), and data were processed using FlowJo software. Fresh mononuclear cells (0.5 × 10^6^) were rested overnight and then stimulated with 1 × 10^6^ K562 cells for 3 h at 37*°*C. Alternatively, mononuclear cells were cultured overnight with 0.5 µg/L IL-12 and then transferred to polystyrene plates coated with anti-NKp46 mAb for 3 h. Cells were stained with mAb to CD3, CD16, CD56, and CD107a. Cells also were fixed in 2% paraformaldehyde solution, then permeabilized (1× Permeabilization buffer, eBioscience) and stained with anti-IFN-γ and anti-MIP-1β mAb.

### Body Composition and Strength Measurements

In cohort D, body composition was measured by dual X-ray absorptiometry (DXA) using a GE Lunar iDXA. Standardized methods for regional partitioning and phantom calibrations were employed to ensure data quality. Scans were analyzed using the GE Lunar software v10.0 in order to calculate fat-free mass (kg), mineral-free lean mass (kg), fat mass (kg), and percent fat. Leanness is a significant risk factor for health outcomes in the elderly (20, 21) and was calculated as appendicular lean mass divided by body mass index (BMI). aLM was calculated as the sum of lean soft tissue in both the right and left arms and legs where limbs were isolated from the trunk by using DXA pre-defined regional lines with manual adjustment. Computed tomography (CT) is described in Supplementary Material.

Measures of strength included isometric and isokinetic knee extension testing on a Biodex System 4 dynamometer. Subjects were given a familiarization training session to acclimate to the testing protocol. During testing sessions, isometric measurements of peak torque and time to peak torque were completed with the subject seated with hip and knee angles at 85° and 90°, respectively. Peak torque was recorded as the highest torque achieved over three trials, whereas time to peak was recorded as the time in seconds to reach peak torque. Knee extensor isokinetic strength testing was completed at 90 degrees per second. We assessed peak torque, time to peak torque, total work, and average power over three trials.

### IL-15 Assay

Plasma IL-15 was measured with the QuantiGlo Chemiluminescent Immunoassay kit (R&D Systems) in two independent experiments, as described in Ref. (19).

### Adipose Tissue Fractionation and Muscle and Adipose Tissue RNA

Subcutaneous adipose tissue and VAT samples were either frozen at −80°C if unfractionated, or immediately processed if they were to be separated into adipocyte and SVF. Unfractionated fat (~200 mg) or muscle (100 mg) were mixed with zirconium oxide beads and 1.0 mL of TRIzol (Thermo-Fisher) in a 1.6 ml microcentrifuge tube (Thermo-Fisher) and treated with a tissue disruptor (Bullet Blender Storm 5, Next Advance) at full speed for 2 min. RNA was purified from the homogenized samples using the RNAeasy Lipid Tissue Mini kit (Qiagen). Nucleic acid (~1.0 μg) was next treated with DNase 1 (Promega). After DNase inactivation, RNA was reverse transcribed using the RNA to cDNA high capacity kit (Thermo-Fisher), following manufacturer protocol.

To prepare adipocyte and SVF fractions, 10–15 g of fresh SAT or VAT was washed thoroughly with Hanks balanced salt solution (HBSS, Sigma), minced, and after clots and connective tissue was removed, subdivided into two 50 mL polypropylene conical tubes. Each tube contained 25 mL of HBSS supplemented with 40 mM HEPES (pH 7.2), 1.0% BSA (Fraction V, Bioworld), 2 mM CaCl_2_ and 0.1% collagenase (Type 1, Worthington, OH, USA). Tubes were gently agitated on an orbital shaker for 3 h at room temperature, centrifuged at 12 × *g* for 5 min, and the floating adipocyte layer and undigested fat was removed. This fraction was sent through a 500 µm steel mesh filter to remove undigested fat and washed once with HBSS. Approximately 0.1–0.2 mL of this fraction was treated with 0.9 mL of TRIzol. The SVF was centrifuged at 300 × *g* and then treated with 1.0 mM EDTA-HBSS in at 37 C for 15 min and centrifuged at 300 × *g*. Pelleted cells were resuspended in HBSS in 1.0 mM EDTA, sent through a 70 µm filter to remove large adipocytes, centrifuged, and solubilized with 1.0 mL of TRIzol. For both adipocytes and SVF, RNA was purified and cDNA prepared as described above.

#### qPCR

Primers and primer design are described in Supplementary Material. We wished to estimate the IL-15 and IL-15Rα RNA that was made by adipocytes and SVF cells. Here, we distinguish contaminating adipocytes from all other SVF cells, which likely includes pre-adipocytes. We first used differentiated adult-derived human adipose stem cells (22) to confirm that adipocytes do not contain CD45 RNA. The SVF CD45 level was only 5% of that of blood mononuclear cells. Using this very conservative 5% level, we estimated the SVF RNA contamination of the adipocyte fraction, which had a median of 2.27%. Based on this measurement, we reasoned that the adiponectin level in the adipocyte fraction would closely estimate the percentage adipocyte RNA. We then used the adiponectin RNA level in the SVF to calculate the percentage of adipocyte RNA, which was a median of 0.5% compared to adipocytes. Using the amount of adiponectin signal in each SFV, we calculated the amount of IL-15 and IL-15Rα RNA that came from SVF cells.

### Statistical Analysis

All statistical tests were run using IBM SPSS Software (version 24, Armonk, NY, USA). The strength and direction of associations were evaluated using the nonparametric Spearman rank-order correlation coefficient (Spearman’s correlation, for short) or the Pearson product-moment correlation coefficient (Pearson’s correlation, for short). Linear regression analysis was used to quantify how well two variables relate to each other. When two or more independent variables were hypothesized to affect the outcome, multiple regression analysis was used. When sample groups were not normally distributed, differences between groups were compared by related samples Wilcoxon Singed Rank Testing. All histogram charts represent single values. Significance was set at <0.05. For box-and-whisker plots, the center lines show the medians; box limits indicate the 25th and 75th percentiles, whiskers extend 1.5 times the interquartile range from the 25th and 75th percentiles. Box and whisker graphs were plotted using BoxPlotR (http://shiny.chemgrid.org/boxplotr/).

## Results

### IL-15 and IL-15Rα RNAs Are Produced in Paired Human SAT and Muscle Samples

**Figure 1:**
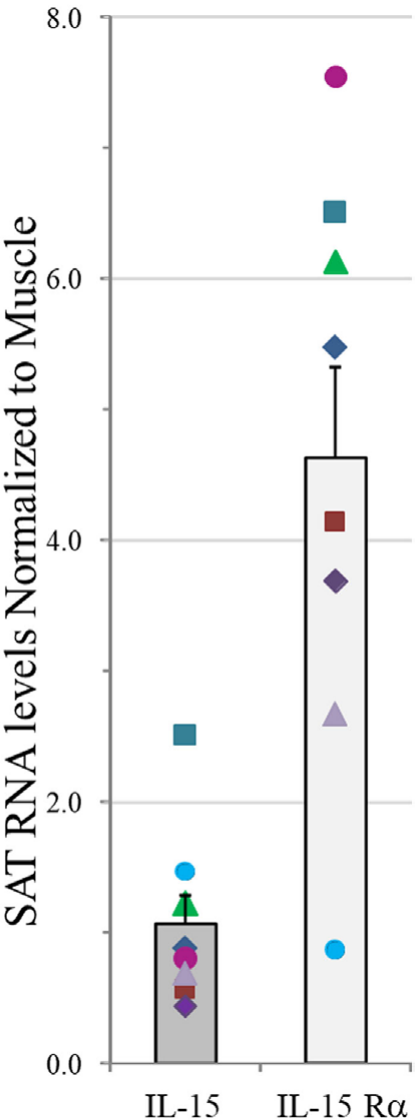
IL-15 and IL-15Rα RNA levels in abdominal subcutaneous adipose tissue (SAT) normalized to values in paired vastus lateralis muscle samples in measured by RT-qPCR and normalized to four housekeeping genes in cohort A. Shown are means plus SEM. Each symbol for IL-15 and IL-15Rα represents an individual donor. IL-15 level did not significantly differ between tissues, but SAT expressed significantly more IL-15Rα RNA than did skeletal muscle (*p* = 0.012). Cohort A is described in Table S1 in Supplementary Material.

Natural killer cell and other innate immune functions decline with age. We hypothesized that IL-15 and its chaperone, IL-15Rα, are secreted from skeletal muscle, SAT, and VAT and influence NK number and function. Therefore, we measured IL-15 and IL-15Rα RNA levels in paired samples of vastus lateralis muscle and abdominal SAT from eight healthy cohort A adult subjects (cohorts are described in Table S1 in Supplementary Material). IL-15 RNA levels in muscle and SAT were not significantly different, but SAT expressed more of the IL-15 chaperone, IL-15Rα. These data are presented as a ratio of SAT RNA level to muscle RNA level in the same individual (Figure 1). This result indicates that fat is a significant source of IL-15 and IL-15Rα RNA and that IL-15Rα RNA level is higher in SAT, compared with skeletal muscle from the same individual.

### IL-15 and IL-15Rα RNAs Are Produced by both VAT and SAT

**Figure 2:**
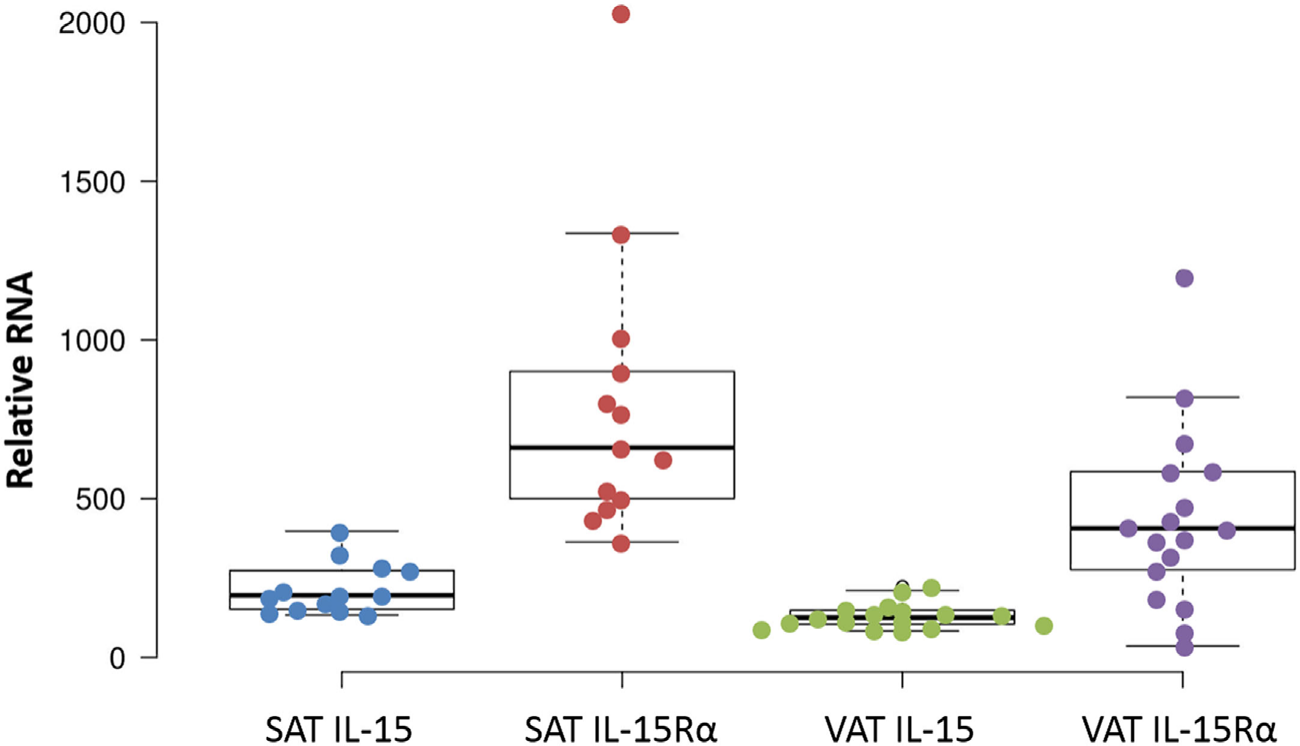
IL-15 and IL-15Rα RNA are produced in both subcutaneous adipose tissue (SAT) and visceral adipose tissue (VAT). Except for one sample, all cohort B SAT samples were from females, from surgeries for panniculectomy (7), ventral hernia (1), breast reduction (4), and mastectomy (1). VAT subjects had surgeries for hernia repair (1), nephrectomy (3), colectomy (12), and gastrectomy (1). Cohort B is described in Table S1 in Supplementary Material.

**Figure 3:**
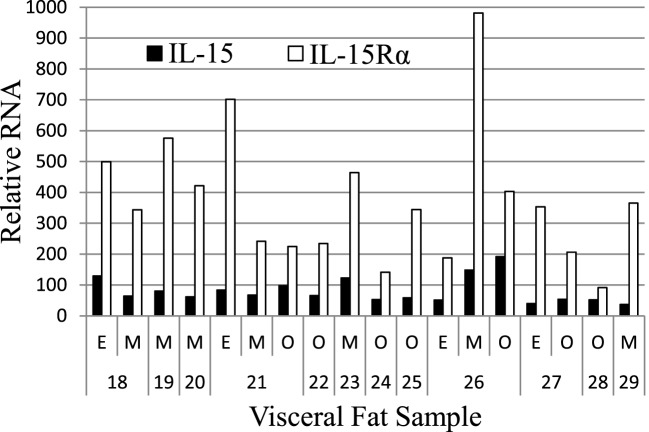
IL-15 and IL-15Rα RNA are expressed in epiploic appendage (E), mesenteric (M), and omental (O) VAT. Cohort C subjects had surgeries for hernia repair (male #22, female #23, male #25), ostomy reversal (male #24), rectal intussusception (female #18), malfunctioning ileal conduit (male #19), large bowel obstruction (female #20), cancer (male #21, male #26, female #27, female #28), and acute appendicitis (male #29). Cohort C is described in Table S1 in Supplementary Material.

We extended these findings by measuring the IL-15 and IL-15Rα transcript levels in SAT and VAT. Non-paired samples of SAT and VAT from surgeries were processed in cohorts B and C. The levels of IL-15 and IL-15Rα transcripts are shown in Figures 2 and 3. As indicated in the figure legend, some VAT samples were from donors with inflammatory conditions (e.g., cancer), but transcript levels from these samples did not differ from donors without inflammatory conditions, such as hernia repair (Figures 2 and 3). Both SAT and VAT produced considerable IL-15 and IL-15Rα RNA (Figure 2). VAT from the mesentery, epiploic appendages, and omentum all produced IL-15 and IL-15Rα RNAs (Figure 3). IL-15 and IL-15Rα RNA levels occasionally differed between VAT depots, but the differences were not found in all subjects. For example, IL-15Rα RNA was higher in epiploic fat than in mesenteric fat in subject 21, but the opposite was true in subject 26 (Figure 3).

### SVF Cells Produced More IL-15 RNA than Did Adipocytes

**Figure 4:**
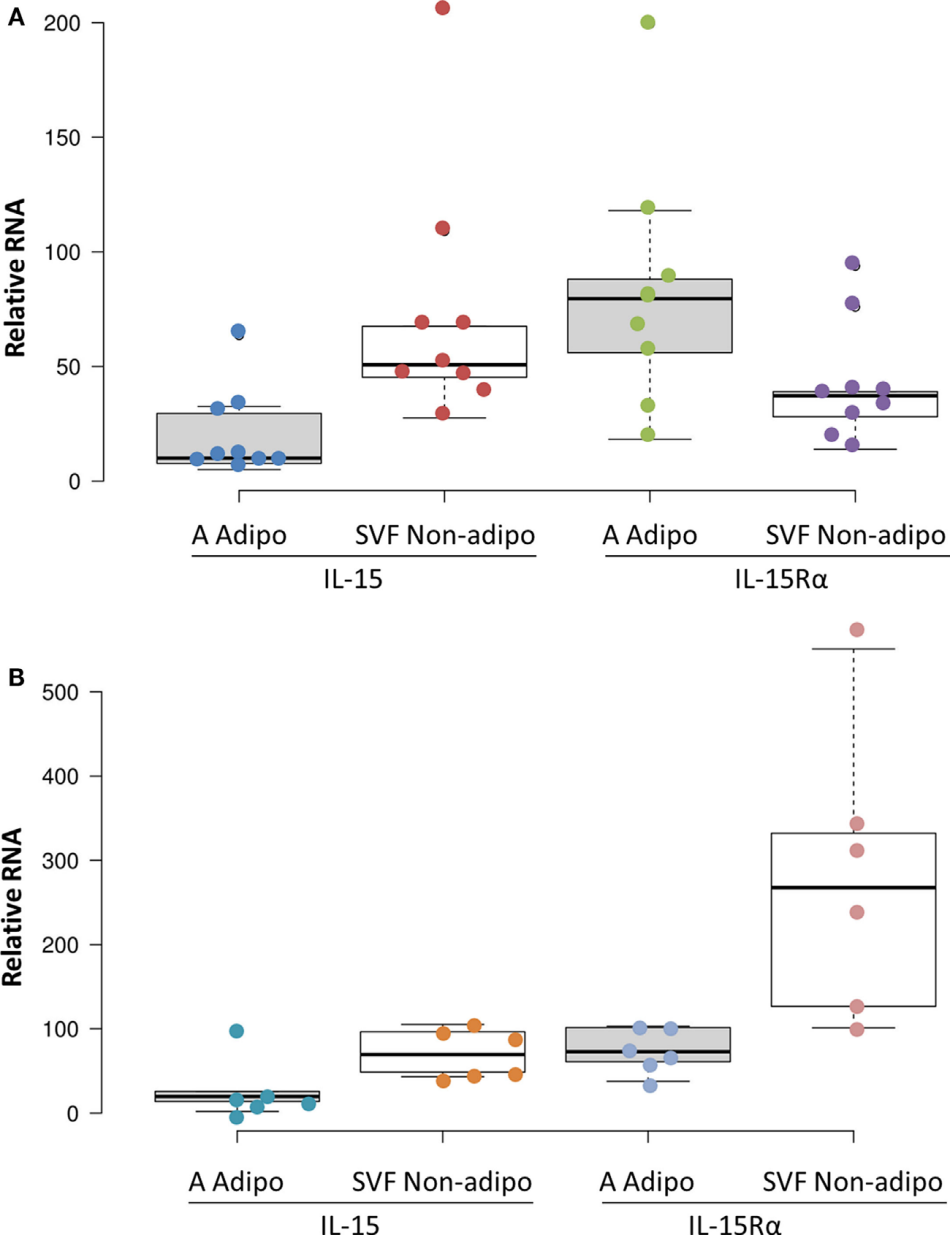
Subcutaneous adipose tissue (SAT) **(A)** and visceral adipose tissue (VAT) **(B)** stromal vascular fraction (SVF) cells produced more IL-15 RNA than did adipocytes (*p* = 0.012 and *p* = 0.043, respectively, by Wilcoxon Signed Rank Test) in cohort B. IL-15Rα RNA levels did not significantly differ between SVF and adipocytes. Adipocyte (A Adipo) and non-adipocyte (SVF Non-adipo) were calculated to exclude contaminating cells as described in Section “Materials and Methods.” All but one SAT samples were from female patients. SAT samples were from breast reductions (5), panniculectomy (4), and ventral hernia repair (1). None of the SAT cases involved cancer or other inflammatory diseases. VAT samples were from colectomies (5), exploratory laparotomy (1), and gastrectomy (1). Two VAT cases did not involve cancer. Cohort B is described in Table S1 in Supplementary Material.

Adipose tissue is comprised of adipocytes, pre-adipocytes, stromal cells, and a variety of leukocytes (23), any one of which could be a source of IL-15 and IL-15Rα. To understand which cells produce IL-15 and IL-15Rα transcripts, fresh SAT and VAT received from surgery were fractionated into adipocyte fraction and SVF. Figure 4 shows that SVF cells expressed more IL-15 RNA than did adipocytes from the same tissue sample. IL-15Rα RNA levels did not differ significantly between the paired samples. From these results, we propose that VAT IL-15 largely comes from SVF cells, which likely include Mφ.

### Plasma IL-15 Level Directly Correlated with VAT

**Figure 5:**
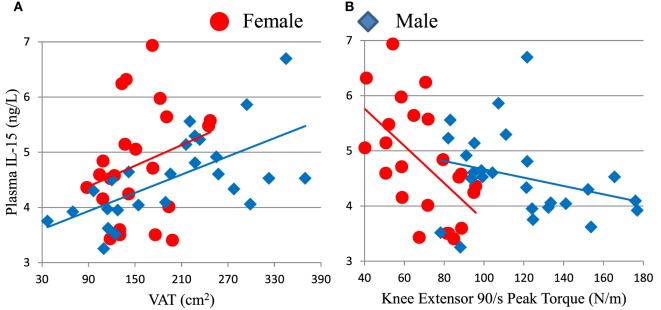
Plasma IL-15 level associates with visceral adipose tissue (VAT) **(A)** and inversely associates with knee extensor peak torque **(B)** in cohort D. Associations of IL-15 with VAT were significant in all subjects (ρ = 0.442, *p* = 0.001) and in males (ρ = 0.657, *p* < 0.001), but not in females tested separately. IL-15 inversely associated with strength in all subjects (ρ = −0.348, *p* = 0.014) and in females (ρ = −0.581, *p* = 0.004), but not in males considered separately. Cohort D is described in Table S1 in Supplementary Material.

We hypothesized that non-lymphoid sources of IL-15 and IL-15Rα may influence plasma IL-15 levels and NK cell activity in the elderly. To test our hypothesis, we recruited 50 healthy adults aged >70 years old and correlated their body composition to plasma IL-15 levels and to NK cell number and function (cohort D). In these elderly subjects, IL-15 plasma levels did not differ by gender (19). IL-15 correlated strongly with CT measures of total abdominal fat and VAT (Figure 5A), but not with abdominal SAT (Table 1). The correlation between IL-15 and VAT was even stronger when analysis was limited to non-obese subjects (BMI < 30; data not shown). Because cytomegalovirus (CMV) infection is life-long and profoundly affects the human immune system (12), we tested whether the correlation between IL-15 and VAT could be explained by CMV infection status. CMV did not correlate with NK cell response to multiple different stimuli (24). Importantly, the correlation between VAT and IL-15 remained strong when CMV status was included as a factor in multifactorial analysis (Table 1). In multifactorial testing, the associations of IL-15 with other adipose depots were not significant when VAT was included as a factor (Table 1). Together, these data indicate that amount of VAT, but not other adipose depots, predicts circulating IL-15 concentration in elderly subjects.

**Table 1:** Correlations with plasma IL-15 level in Cohort D.^a^

	Parameter	Spearman	Reg. (age, sex)^b^	Reg. [visceral adipose tissue (VAT)]^c^	Reg. (torque)^d^	Reg. [cytomegalovirus (CMV)]^e^
Computed tomography (CT) fat	Abdomen	0.391, 0.005	0.325, 0.027	0.181, 0.336		
	Abdominal subcutaneous adipose tissue	0.171, 0.241	0.156, 0.355	0.135, 0.327		
	VAT	0.442, 0.001	0.450, 0.002		0.423, 0.001	0.372, 0.007

Dual X-ray absorptiometry fat	Total	0.334, 0.018	0.286, 0.053	0.160, 0.317		
	Legs	0.232, 0.105	0.125, 0.360	0.165, 0.232		
	Trunk	0.342, 0.015	0.334, 0.020	0.117, 0.507		
	Android	0.302, 0.033	0.327, 0.019	0.047, 0.805		

Other	Appendicular lean mass divided by BMI	−0.344, 0.014	−0.743, 0.001	−0.388, 0.003	−0.313, 0.100	
	Muscle area^f^	−0.244, 0.091	−0.155, 0.394	−0.417, 0.004	0.030, 0.860	
	Knee torque	−0.348, 0.014	−0.598, 0.021	−0.397, 0.002		−0.352, 0.010

*^a^The correlation coefficient and the significance are show in each cell. Significant associations are underlined*.

*^b^Linear regression corrected for influence of age and gender*.

*^c^Linear regression corrected for influence of VAT*.

*^d^Linear regression corrected for influence of knee extensor peak torque*.

*^e^Linear regression, corrected for influence of CMV infection*.

*^f^CT-measured thigh muscle cross-sectional area*.

### The Elderly Subject Cohort D Showed Expected Sex Differences and Had Low C-Reactive Protein Level

As expected, men had significantly greater muscle mass, bone mineral content, fat-free mass, and android fat, whereas women had more gynoid and leg fat by DXA and more SAT by CT (data not shown). Men had more VAT than women by CT, but this difference was not significant. For all subjects, C-reactive protein levels were <10 mg/L, indicating a lack of marked inflammatory disease (data not shown). As expected, C-reactive protein level correlated with measures of adipose tissue and inversely with leanness. C-reactive protein correlated directly with IL-15 (data not shown), suggesting a link between systemic inflammation and circulating IL-15 level.

### NK Cell Activity Directly Correlated with IL-15 Level

To investigate whether or not plasma IL-15 correlated with NK cell activity, we stimulated elderly cohort D peripheral blood mononuclear cells *in vitro* with K562 leukemia cells or with low-level IL-12 and immobilized anti-NKp46 antibody, which are well-known NK cell stimuli. Using flow cytometry, we measured the ability of CD56^bright^ and CD56^dim^ NK cells to produce IFN-γ and MIP-1β. We also measured their cytotoxic response, as assayed by the appearance of the CD107a cytotoxic granule marker on the cell surface. Plasma IL-15 level correlated with the CD56^dim^ NK cell MIP-1β and CD107a, but not IFN-γ, responses to NKp46 crosslinking (Table S2 in Supplementary Material). The correlations between NKp46-stimulated responses and plasma IL-15 level remained significant when correcting for age and gender. The responses to NKp46 did not significantly correlate with the responses to K562 leukemia cells, indicating that these two assays measure distinct aspects of NK cell signaling (data not shown). This is not surprising because NK cell responses to K562 largely depend upon NKp30 and NKG2D (25).

### NK Cell Activity Inversely Correlated with Muscle Strength

**Figure 6:**
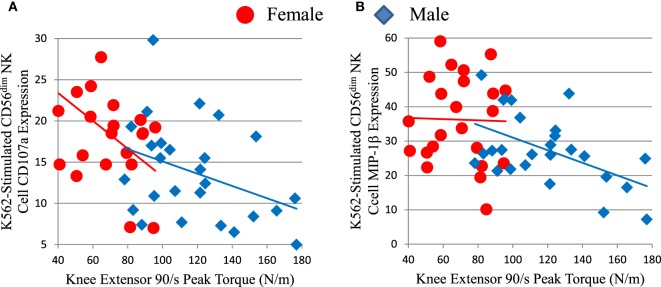
Strength (knee extensor peak torque) correlates inversely with measures of mature CD56^dim^ natural killer (NK) cell responses to K562 leukemia cells in cohort D. CD56^dim^ NK cell CD107a **(A)** is a measure of cytotoxic granule release and correlated inversely with peak torque for all subjects (ρ = −0.475, *p* = 0.001), but not for each sex considered individually. CD56^dim^ NK cell MIP-1β chemokine production **(B)** correlated inversely with peak torque for all subjects (ρ = −0.384, *p* = 0.006), but not for each sex considered individually.

We compared NK cell responses and muscle strength. Mature CD56^dim^ NK cell responses to K562 leukemia cells showed inverse correlations with muscle strength, as measured by knee extensor peak torque (Figure 6; Table S2 in Supplementary Material), isometric peak torque, and average power (data not shown). Both CD56^dim^ NK cell responses to K562 target cells were significant or trended against strength after factoring in age and sex (Table S2 in Supplementary Material). As expected, skeletal muscle mass correlated with strength in this elderly cohort D (data not shown). Notably, both CD56^dim^ NK cell degranulation (as measured by CD107a) and MIP-1β responses to K562 cells robustly inversely associated with strength after factoring in either thigh muscle mass or leanness (Table S2 in Supplementary Material and data not shown). This indicates that muscle quality substantially and inversely associated with NK cell response to leukemia cells. As with other associations, CMV status did not weaken this inverse correlation (Table S2 in Supplementary Material).

Interestingly, SAT, but not VAT, correlated with the density of CD38 expression on both CD56^bright^ and CD56^dim^ NK cells (Table S3 and Figure S1 in Supplementary Material), which is described and discussed in Supplementary Material.

### Strength Inversely Correlated with Plasma IL-15 in Elderly Humans

Because IL-15 is anabolic for skeletal muscle in some situations and skeletal muscle itself may be a source of IL-15, we predicted that skeletal muscle and plasma IL-15 would directly correlate (18). However, there was no significant association between skeletal muscle mass and plasma IL-15 (Table 1). Because muscle mass declines less rapidly in elderly people than does strength, we searched for an association between strength and IL-15. We found a significant inverse correlation between IL-15 and knee extensor peak torque (Table 1; Figure 5B). This inverse association was confirmed when sex and age were included in multifactorial analysis; the inverse association became even more robust when VAT was included as a factor in multifactorial analysis (Table 1), indicating that this correlation is not confounded by VAT volume. Likewise, the correlation remained strongly negative when a role for CMV infection status was tested, suggesting that this correlation is not dependent on CMV status. When both knee extensor peak torque and thigh muscle cross-sectional area were compared, muscle strength, but not muscle cross-sectional area, was significant (Table 1).

## Discussion

The objectives of this work were to determine (1) if human muscle, SAT, and VAT are sources of IL-15 and IL-15 Rα, and (2) whether any of these tissues correlate with NK cell activity in elderly humans. We found that skeletal muscle, SAT, and VAT all produced IL-15 and IL-15Rα RNA. Of these, only VAT correlated with IL-15 plasma levels in elderly human subjects. Our findings suggest that VAT may support NK homeostasis and activity in the elderly, when IL-15-producing immune cells have declined.

**Figure 7:**
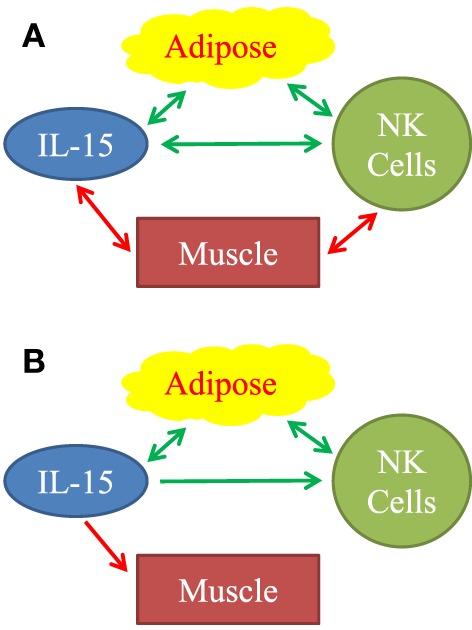
Observed associations **(A)** and proposed mechanistic relationships **(B)** between adipose tissue, IL-15, natural killer (NK) cells, and muscle. **(A)** Positive associations are represented by green arrows. Inverse associations are represented by red arrows. All arrows are double-headed, indicating that associations do not necessarily imply mechanism. Mechanistic model **(B)** is based on our findings and on published literature. Stimulatory mechanisms are represented by green arrows and inhibitory mechanisms are represented by the red arrow. Double-headed green arrows indicate mutually positive stimulation. We propose that adipose tissue is a significant source of IL-15, especially in aging humans. IL-15 stimulates NK cells. Importantly, IL-15 forms part of a positive feedback loop between adipose tissue NK cells, other type 1 innate lymphocytes, and macrophages, as represented by the double-headed green arrows. IL-15 and other inflammatory agents weaken muscle.

Other studies indicate that adipose tissue may be a significant source of IL-15. Dozio et al. (26) found that mouse epicardial fat, a type of VAT, expressed IL-15 and IL-15Rα RNA. Hickner and co-workers found that human abdominal SAT and skeletal muscle produce IL-15; and that SAT interstitial IL-15 level was higher in obese vs. lean young adults (27). Like us, this group found IL-15 blood levels did not correlate with total body mass (27). Another group found that IL-15 level was higher in VAT tissue homogenates from obese vs. lean middle-aged people (28). Liou et al. (29) showed that mouse adipocytes make significant amounts of IL-15 and that optimal NK cell development required adipocyte IL-15. Using parabiotic mouse pairs, O’Sullivan found that NK cells recirculate between the blood and the VAT compartments (30). This indicates that circulating NK cells are exposed to cytokines in the VAT (Figure 7B). Christiansen et al. found that young adult subjects placed on 12-week regimes of reduced caloric intake had significant IL-15 *declines* (31). This study suggests that negative caloric balance lowers IL-15 level and because caloric restriction usually leads to loss of fat, is consistent with our finding that increased VAT directly correlated with plasma IL-15 level (Figure 7B).

We attempted to determine if human skeletal muscle and fat derived IL-15 and IL-15Rα may correlate with NK activity in the elderly. Definitive experimental manipulation of human subjects is not possible, but we identified strong correlations between body composition parameters, IL-15 plasma levels, and NK cell function. We restricted our main analysis to elderly people because a prior study had suggested a correlation between BMI and NK cell number in elderly women, but not in young women (18). Additionally, because excess VAT and SAT carry different health risks, we separately analyzed correlations with IL-15 and NK cell activity. Our data, summarized graphically in Figure 7A, suggests that non-lymphoid tissues affect NK cells *via* multiple distinct interactions, but the strongest direct correlations were between IL-15 plasma levels and VAT, and an inverse correlation between NK function and muscle strength.

Our study appears to contrast with several studies of rodents and humans in which serum IL-15 level negatively associated with VAT (32–37). As mentioned above, we found that plasma IL-15 positively associated with VAT. It is useful to consider that abdominal VAT may respond to IL-15 and that VAT itself produces IL-15 (27, 29). Most of the studies in rodents involved animals exposed to high nonphysiologic IL-15 levels *via* transgene expression or *via* injection; other studies utilized mice that were IL-15 knockouts. These extremes of IL-15 exposure might not be good models of the human condition. Prior human studies involved young adults and sometimes obese and lean groups differed significantly in age (33, 38). The contrasting outcomes in our study and past human studies may reflect age-related physiological differences. One effect of IL-15 is to increase gene expression and metabolic activity in brown adipose tissue (39). Because the amount of brown adipose tissue usually declines with age (40), many elderly individuals might not respond to IL-15 by increasing brown fat metabolic activity. Another possible explanation for this apparent contradiction is the amount of skeletal muscle mass in young and elderly adults and its correlation with adipose tissue mass. Obese young adults typically have more muscle mass than do lean young adults (41). Yet, fat mass is associated with a more rapid decline in muscle mass during aging (41). In addition, muscle strength declines rapidly in the elderly, much faster than loss of muscle mass, and may be a better measure of skeletal muscle health and function (42, 43).

Macrophages and DC functions decline with aging (13, 15, 44), making non-lymphoid sources of IL-15 relatively more important. These factors are likely to influence IL-15-depedent NK cells and memory CD8 T cells in the elderly. We propose that in elderly humans, VAT is a significant incremental source of IL-15 (Figure 7B) and promotes NK cell homeostasis.

Growing evidence supports the hypothesis of a positive feedback loop between type 1 innate lymphoid cells (including NK cells) and Mφ in people with a positive energy balance (30, 45–47). Adipocyte hypertrophy, fibrosis, hypoxia, and cell death cause release of inflammatory molecules, which stimulate adipose tissue Mφ (48). Stressed adipocytes express NKp46 ligands that directly stimulate NK cells and probably other type 1 innate lymphoid cells (47). In response to the inflammatory molecules, Mφ produce IL-12 and IL-15, which stimulate NK cells and other type 1innate lymphoid cells to produce IFN-γ, TNFα, and IL-6 (30, 45). These products, in turn, stimulate Mφ, setting up a positive feedback loop. Inflammatory cytokines, including IL-6 and TNFα, which affect skeletal muscle, the vasculature, and other tissues, cause pathologies associated with frailty and the metabolic syndrome (48, 49). Our data fit into this picture (Figure 7B). We found that IL-15 is elevated in relation to human VAT mass. Plasma C-reactive protein, a measure of inflammation, correlated strongly with plasma IL-15 (data not shown). We propose that IL-15 stimulates NK cells, both in a local paracrine fashion in VAT and in an endocrine fashion (Figure 7B). To explain the negative correlation between IL-15 and muscle strength, we propose that IL-15 itself and other inflammatory factors inhibit skeletal muscle (Figure 7B).

## Ethics Statement

In accordance with the Declaration of Helsinki (modified in 2008), all protocols were approved by the Institutional Review Board of the University of Kentucky, Lexington, KY, USA. All subjects were made aware of the design and purpose of the studies, and all subjects signed consent forms. The cohorts are summarized in Table S1 in Supplementary Material, including IRB approval numbers, where applicable.

## Author Contributions

AA-A, SP, JC, DL, and RW performed experiments, analyzed data, and edited manuscript. MSexton and MS performed experiments and edited manuscript. PK, CP, and CL analyzed data and edited manuscript.

## Conflict of Interest Statement

The authors declare that the research was conducted in the absence of any commercial or financial relationships that could be construed as a potential conflict of interest.

## Abbreviations

aLM/BMI, appendicular lean mass divided by BMI; BMI, body mass index; CT, computed tomography; DC, dendritic cell; DXA, dual X-ray absorptiometry; MIP-1β, chemokine CCL4; Mφ, macrophage; SAT, subcutaneous adipose tissue; SVF, stromal vascular fraction; VAT, visceral adipose tissue.
