# Supplementary material for: Human Body Composition and Immunity: Visceral Adipose Tissue Produces IL-15 and Muscle Strength Inversely Correlates with NK Cell Function in Elderly Humans
Source: Front Immunol. 2018 Mar 6;9:440. doi: 10.3389/fimmu.2018.00440 (PMC5845694; doi:10.3389/fimmu.2018.00440)
Supplement: Supplementary file 1 [file Presentation_1.PDF]

Supplementary Methods.

Computed tomography (CT) measured adipose and skeletal muscle cross-sectional areas (cm^2) of the upper leg and abdomen in all but one female subject and were performed using a Siemens Definition AS CT scanner (Holland, MI) with 140 kV and a slice thickness of 5 mm using previously described methodology (1). Briefly, the subjects were clothed only in a loose gown and examined in a supine position with their arms stretched above their heads. An abdominal scan at the level of the L4 intervertebral space was performed with no angulation with a lateral pilot for location, and a second CT scan performed at midthigh, midpoint between the inguinal crease and the superior border of the patella. Abdominal VAT, abdominal SAT, and total abdominal fat cross-sectional areas (cm^2) were calculated by delineating, with a mouse computer interface, the designated areas and then computing the adipose tissue using an attenuation range from -190 to -30 HU using NIH ImageJ version 1.34e software (htttp://rsbweb.nih.gov/ij/). Similarly, the thigh CT scans were analyzed to determine total thigh adipose tissue and skeletal muscle cross-sectional area using attenuation range from 0 to 100 HU. In addition, the skeletal muscle will be subdivided into areas of low attenuation (0-30 HU) representing "fat-rich" muscle, and high attenuation values (31-100 HU) representing density muscle with normal fat content.

qPCR. The primer sequences (IDT technologies, or Sigma) in the 5' to 3' direction were adiponectin: forward GGT CAT GAC CAG GAA ACC AC, reverse TTC ACC GAT GTC TCC CTT AGG; CD45: forward AAC AAC CAC TCT GAG CCC TTC T, reverse CCCCTGGTGGCACATCTAATA; PGK-1 forward GCT GGA CAA GCT GGA CGT TA, reverse AGC AGC CTT AAT CCT CTG GT; SDHA forward TGG GAA CAA GAG GGC ATC TG, reverse CCA CCA CTG CAT CAA ATT CAT G; INTS4: forward GCA GCT CCA TGA AAG AGG AC, reverse ACC CAG ATA AGC TGG ACT GC; RPLP0: forward GGC GAC CTG GAA GTC CAA CT, reverse CCA TCA GCA CCA CAG CCT TC; IL-15: forward CTA ACT GAA GCT GGC ATT CAT GT, reverse ACT TAT TAC ATT CAC CCA GTT GGC, hydrolysis probe: 5,6-FAM-TAG GAA GCC CTG CAC TGA AAC AG –IaBk-3', IL- 15 Ra forward TAT CTC CAC GTC CAC TGT C, reverse TCC ATG GCT TCC ATT TCA AC, hydrolysis probe 5,6-FAM-CCT GGC ATG CTA CCT CAA GTC AA–IaBk-3'. All primers had an amplification efficiency measured to be between 90 and 110%, and agarose gel analysis confirmed that amplicons were the correct size. The program used for adiponectin, CD45, INTS4, RPLP0, SDHA, and PGK-1 transcript detection was denaturation at 95.0°C for 5 sec, annealing at 66.0°C for 10 sec, and extension at 72°C for 30 sec, using 1x SYBR green mix (Bioline). For IL-15 and IL-15Rα, denaturation was 95.0°C for 10 sec, and annealing/extension at 60.0°C for 30 sec using 1x PerfeCTa qPCR fast mix UNG (Quanta Bio). All sample volumes were 10 uL and amplified in triplicate reactions. C_q was determined from amplification curves using Bio-Rad CFX manager software. The fold change of adiponectin, CD45, IL-15, and IL-15Rα transcript levels compared to the geometric mean of four housekeeping (HK) gene RNAs (INTS4, RPLP0, SDHA, and PGK-1) was calculated using 2^(-($C_{q\ target} – C_{q\ geo\ mean\ HK}$).

Supplementary Table S1: Experimental Subjects

Cohort	Demographics	Exclusions	Reference	IRB Approval
A	8 healthy subjects, 1 ♂, BMI 24.1 – 36.1, insulin sensitivity 1.28-6.81, ages 28-64 years (mean 52.8)	history of smoking, coronary disease, congestive heart failure, chronic inflammatory diseases, hypertriglyceridemia, certain orthopedic problems	3	09-0853
B	17 surgery patients. 10 (1 ♂) for SAT, ages 24-62 years (mean 42.5) and 7 (4 ♂) for VAT, ages 54-71 years (mean 62.1).	SAT: Cancer or inflammatory diseases; VAT: cancer near the VAT; two did not have cancer.		De-identified samples, not human subjects research
C	12 surgery patients (7 ♂), ages 25-75 years (mean 56.6).	VAT: cancer near the VAT; eight did not have cancer, one had acute appendicitis. BMI > 35.5, immunosuppressive drugs within 48 hr of surgery, HIV.		12-0004-F2L
D	50 healthy subjects, 26 ♂, > 70 years old (mean ♂ 77.8, ♀ 77.0). ♂ BMI 21.8-41.2, mean 27.8. ♀ BMI 20.3-35.2, mean 27.3.	Immune-related diseases, immune suppression drugs, diabetes, acute illness in the preceding week.	2	11-0771-F1V

Supplementary Results and Discussion. The most important correlations between body composition, plasma IL-15 levels, and NK activity are mentioned in the main text. However, we note the following:

The inverse correlation of plasma IL-15 and strength (Fig. 5B) parallels the inverse correlation of strength and $CD56^{dim}$ NK cell activity (Fig. 6A and 6B), and the direct correlation of plasma IL-15 and mature $CD56^{dim}$ NK cell activity (Supplementary Table S2). It suggests that IL-15 directly benefits NK cells and further suggests that IL-15 could weaken skeletal muscle, probably in concert with other inflammatory factors.

Despite IL-15 level correlating positively with both VAT and $CD56^{dim}$ NK cell activity (Fig.5A and Supplementary Table S2), there was no significant correlation between VAT and any NK cell parameter when factoring in age and gender. This suggests that many factors control VAT, IL-15 level, and NK cell activation. Many factors rise with increased adipose mass, including leptin, chemerin, resistin, TNF-α, IL-1, and IL-6 (2, 3).

Supplementary Table S2: Correlations with CD56^{dim} NK cell function*

Correlate	Stimulus	Analyte	Spearman	Reg. (age, sex)**	Reg. (SkM)†	Reg. (CMV)‡
IL-15	K562	CD107a	.368, .009	.252, .063		
		MIP-1β	.279, <.05	.194, .167		
	NKp46	CD107a	.314, .026	.306, .036		
		MIP-1β	.306, .031	.304, .030		
aLM/BMI	K562	CD107a	-.366, .009	-.244, .280		
		MIP-1β	-.292, .040	-.181, .434		
Knee torque	K562	CD107a	-.457, .001	-.420, .082	-.521, .002	-.490, .000
		MIP-1β	-.384, .006	-.490, .048	-.436, .011	-.449, .001

* The correlation coefficient and the significance are shown in each cell in cohort D. Significant associations are underlined.

** Linear regression, corrected for influence of age and gender.

† Linear regression, corrected for influence of right upper leg skeletal muscle area as assessed by CT scan.

‡ Linear regression, corrected for influence of CMV infection

CD38, an activation marker on NK cells and other lymphocytes, strongly correlated with many parameters of SAT (Supplementary Table S3 and Supplementary Fig. S1). This result is unique in our study, in the respect that all other significant correlations between NK surface markers or activity occurred with VAT, and not SAT. This suggests that although VAT and SAT share many properties, they differentially secrete molecules that affect NK cells, and therefore the immune system.

Supplementary Table S3:
SAT, but not VAT, correlates with both CD56^{bright} and CD56^{dim} NK cell CD38 density *

	Adipose	Spearman	Reg. (age, sex)**	Reg. (CMV)
CD56^{bright}	DXA Total	.366, .011	.354, .013	.359, .014
	DXA Legs	.469, .001	.406, .009	.457, .002
	DXA Gynoid	.494, .000	.494, .001	.532, .000
	CT Thigh	.528, .000	.610, .000	.590, .000
	CT Abdomen	.484, .001	.376, .009	.406, .004
	CT Abdominal SAT	.512, .000	.517, .001	.516, .000
	CT VAT	.099, .518		
CD56^{dim}	DXA Total	.328, .021	.345, .013	.375, .008
	DXA Legs	.411, .003	.365, .019	.412, .004
	DXA Gynoid	.450, .001	.492, .001	.524, .000
	CT Thigh	.390, .007	.411, .016	466, .001
	CT Abdomen	.425, .003	.407, .004	.455, .001
	CT Abdominal SAT	.583, .000	.485, .002	.526, .000
	CT VAT	.001, .995		

* The correlation coefficient and the significance are shown in each cell in cohort D. Significant associations are underlined.
** Linear regression, corrected for influence of age and gender.

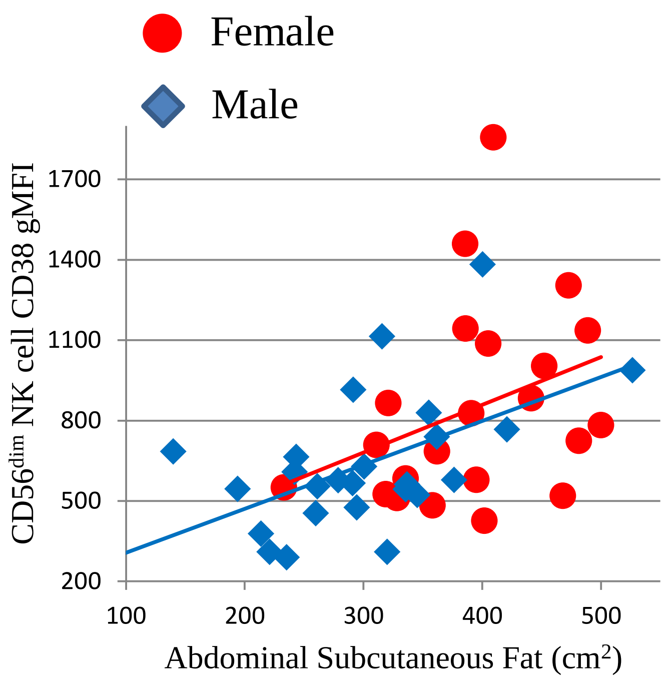

Supplementary Figure S1: CD56^{dim} NK cell CD38 cell surface density correlates with abdominal SAT, in the elderly cohort D.

CD38 is widely regarded as a marker of highly activated T cells (4-7). However, CD38 expression and ectoenzyme activity on the CD56^{bright} NK cell surface allows an enzymatic cascade leading to production of extracellular adenosine (8). CD56^{bright} NK cells use CD38 and adenosine to suppress CD4 T cell proliferation (8). In cohort D, both CD56^{bright} and CD56^{dim} NK cell CD38 density correlated strongly with SAT (Supplementary Table S3, Fig. S1). There was absolutely no correlation with VAT. We observed these correlations (positive with SAT and none with VAT) also with CD4 and CD8 T lymphocyte CD38 expression (data not shown). A number of studies have linked CD38 to adipose tissue and obesity. CD38 deficient mice are resistant to obesity induced by high fat diet; drug inhibition of CD38 lowered liver triglycerides (7, 9). Similar to our findings, CD38 expression on peripheral blood lymphocytes (mostly T cells) was reported to be higher in obese adults than in lean adults (10).

References

1. Clasey, J. L., C. Bouchard, L. Wideman, J. Kanaley, C. D. Teates, M. O. Thorner, M. L. Hartman, and A. Weltman. 1997. The influence of anatomical boundaries, age, and sex on the assessment of abdominal visceral fat. *Obes Res* 5: 395-401.
2. Lutz, C. T., and L. S. Quinn. 2012. Sarcopenia, obesity, and natural killer cell immune senescence in aging: Altered cytokine levels as a common mechanism. *Aging (Albany NY)*.
3. Cesari, M., S. B. Kritchevsky, R. N. Baumgartner, H. H. Atkinson, B. W. H. J. Penninx, L. Lenchik, S. L. Palla, W. T. Ambrosius, R. P. Tracy, and M. Pahor. 2005. Sarcopenia,

obesity, and inflammation--results from the Trial of Angiotensin Converting Enzyme Inhibition and Novel Cardiovascular Risk Factors study. *Am J Clin Nutr* 82: 428-434.

4. Read, S., S. Mauze, C. Asseman, A. Bean, R. Coffman, and F. Powrie. 1998. CD38+ CD45RB(low) CD4+ T cells: a population of T cells with immune regulatory activities in vitro. *European journal of immunology* 28: 3435-3447.
5. Bahri, R., A. Bollinger, T. Bollinger, Z. Orinska, and S. Bulfone-Paus. 2012. Ectonucleotidase CD38 demarcates regulatory, memory-like $CD8^+$ T cells with IFN-γ-mediated suppressor activities. *PloS one* 7: e45234.
6. Chen, J., Y. G. Chen, P. C. Reifsnyder, W. H. Schott, C. H. Lee, M. Osborne, F. Scheuplein, F. Haag, F. Koch-Nolte, D. V. Serreze, and E. H. Leiter. 2006. Targeted disruption of CD38 accelerates autoimmune diabetes in NOD/Lt mice by enhancing autoimmunity in an ADP-ribosyltransferase 2-dependent fashion. *Journal of immunology* 176: 4590-4599.
7. Barbosa, M. T., S. M. Soares, C. M. Novak, D. Sinclair, J. A. Levine, P. Aksoy, and E. N. Chini. 2007. The enzyme CD38 (a NAD glycohydrolase, EC 3.2.2.5) is necessary for the development of diet-induced obesity. *FASEB J* 21: 3629-3639.
8. Morandi, F., A. L. Horenstein, A. Chillemi, V. Quarona, S. Chiesa, A. Imperatori, S. Zanellato, L. Mortara, M. Gattorno, V. Pistoia, and F. Malavasi. 2015. $CD56^{bright}CD16^{-}$ NK cells produce adenosine through a CD38-mediated pathway and act as regulatory cells inhibiting autologous CD4+ T cell proliferation. *Journal of immunology* 195: 965-972.
9. Escande, C., V. Nin, N. L. Price, V. Capellini, A. P. Gomes, M. T. Barbosa, L. O'Neil, T. A. White, D. A. Sinclair, and E. N. Chini. 2013. Flavonoid apigenin is an inhibitor of the NAD+ ase CD38: implications for cellular NAD+ metabolism, protein acetylation, and treatment of metabolic syndrome. *Diabetes* 62: 1084-1093.
10. Lynch, L. A., J. M. O'Connell, A. K. Kwasnik, T. J. Cawood, C. O'Farrelly, and D. B. O'Shea. 2008. Are natural killer cells protecting the metabolically healthy obese patient? *Obesity* 17: 601-605.
